# Elevated temperature and decreased salinity impacts on exogenous *Vibrio parahaemolyticus* infection of eastern oyster, *Crassostrea virginica*

**DOI:** 10.3389/fmicb.2024.1388511

**Published:** 2024-07-04

**Authors:** Omario M. A. Ricketts, Sean R. Isaac, Rhiannon A. Lara, Tyler S. Mendela, Laura A. Enzor, Adam C. Silver

**Affiliations:** Department of Biology, University of Hartford, West Hartford, CT, United States

**Keywords:** *Vibrio parahaemolyticus*, eastern oyster, *Crassostrea virginica*, climate change, elevated temperature, decreased salinity

## Abstract

Anthropogenic carbon emissions have resulted in drastic oceanic changes, including increased acidity, increased temperature, and decreased salinity. Anthropogenic carbon emissions have resulted in drastic oceanic changes, including increased acidity, increased temperature, and decreased salinity. Few studies have directly assessed the compounded impact of alterations to oceanic conditions on oyster physiology and the relation to the presence of *V. parahaemolyticus*. This project investigated the relationship between projected climate scenarios and their influence on both eastern oyster, Crassostrea virginica, and the aquatic bacteria, Vibrio parahaemolyticus. Specifically, we examined whether an increase in water temperature and/or decrease in salinity would impair oyster resistance to *V. parahaemolyticus*, a human food and waterborne pathogen. Using a culture-dependent approach, our data revealed that the alterations in environmental conditions did not significantly impact the numbers of *V. parahaemolyticus* numbers within oyster hemolymph or tissues. However, we did observe a dramatic increase in the total amount of bacteria and pathogenic native *Vibrio* species, *Vibrio aestuarianus* and *Vibrio harveyi.* Despite detecting *V. parahaemolyticus* in most tissues at 7 days post-challenge, oysters were able to reduce bacterial levels below our limit of detection by 28 days of exposure. Furthermore, in our second experimental trial exploring single vs. multiple inoculation of bacteria, we observed that oysters were either able to reduce total bacterial levels to pre-treatment burdens (i.e., below our limit of detection) or die. This study demonstrates that the synergistic effects of elevated temperature and decreased salinity do not inhibit oysters from preventing the long-term colonization of exogenous *V. parahaemolyticus*. However, our data do show these environmental stressors impact oyster physiology and the native microbiota. This can lead to the proliferation of opportunistic pathogens, which could have impacts on oyster population numbers and ecosystem and human health.

## Introduction

The eastern oyster, *Crassostrea virginica,* is an ecologically important keystone species to estuarine environments. Oyster reefs offer a habitat for various aquatic animals, protect from coastal erosion, and improve water quality and clarity (Kennedy, 1996). Additionally, oysters are considered a highly valued commercial fishery and aquaculture species. In 2020, approximately 42 million pounds of oysters were harvested for human consumption along the U.S. Atlantic Coast, netting over $134 million (NMFS, 2022). As suspension filter feeders, oysters are constantly subjected to several microorganisms that are typically found in seawater (Almuhaideb et al., 2020). These microorganisms not only impact oyster health, but the raw consumption of oysters also creates a common mode of human infection, resulting in increased incidences of gastroenteritis, which can be caused by various *Vibrio* spp., such as *Vibrio parahaemolyticus, Vibrio vulnificus,* and *Vibrio cholera* (FDA, 2012).

*Vibrio parahaemolyticus* is a Gram-negative bacterium that is frequently found in marine and estuarine environments. It is a common cause of bacterial gastroenteritis in Asia; it is the leading cause of seafood-borne illnesses worldwide, and infection numbers have been increasing in the United States (Klein et al., 2018; Park et al., 2018). While it is most notably associated with the consumption of raw shellfish, such as oysters and mussels, *V. parahaemolyticus* can also cause soft-tissue infections if open wounds are exposed to coastal seawater (Klein et al., 2018; Park et al., 2018). Of the estimated 80,000 cases of vibriosis each year in the United States, the CDC predicts *V. parahaemolyticus* is responsible for approximately 45,000 of those illnesses. The pathogenicity of *V. parahaemolyticus* is attributed to different virulence factors, including the motility, ability to form biofilms, and enterotoxins secreted directly into host cells via a type III secretion system (Letchumanan et al., 2014; Raghunath, 2014). *V. parahaemolyticus* genes *tdh* and *trh* encode thermostable direct hemolysin (TDH) and TDH-related hemolysin, respectively, that are responsible for hemolytic, cytotoxic, and enterotoxin activities.

To combat these microbial threats, oysters possess hemocytes (phagocytic immune cells of invertebrates) and immune proteins (e.g., antimicrobial peptides, lysozyme, and lectins) (Cheng, 1975). However, several studies have shown that exposure to environmental factors such as heavy metals, elevated temperature, decreased pH, decreased salinity, and/or decreased dissolved oxygen levels can impair the immune function of oysters, making them more susceptible to disease (Willson and Burnett, 2000; Goedken et al., 2005; Macey et al., 2008; Ivanina et al., 2011; Wendling and Wegner, 2013; Keppel et al., 2015). Not only does increasing temperature negatively impact oyster immune function but it is also linked to a rise in bacterial burdens in aquatic environments, which synergistically could exacerbate the process of both causing disease in oysters and transmitting pathogens to humans via oyster consumption (Froelich B and Oliver JD, 2013; Davis et al., 2017; Almuhaideb et al., 2020). Environmental variables change concomitantly; the interaction of these variables is frequently what drives an organisms’ response (Sokolova, 2013). Climate change models project continued increase in water temperatures and decreased salinity of estuarine waters in the northeastern United States (IPCC, 2014; Easterling et al., 2017; Huang et al., 2017).

Given the roles eastern oysters fulfill as both a keystone species in the estuary and a focal aquaculture species, it is vital to explore how factors of climate change and bacterial exposure impact oyster health. The study aimed to assess how elevated temperature combined with decreased salinity affects the ability of oysters to resist *V. parahaemolyticus* (a human and oyster pathogen) infection and their physiological equilibrium. We built a recirculating exposure system that allowed us to alter temperature and/or salinity levels to reflect their projected levels by the year 2,300 (IPCC, 2014) and inoculate water with bacterial numbers that are comparable to those oysters would encounter in their natural habitat and mimic the natural route of infection. We determined *V. parahaemolyticus* and total culturable bacterial levels in oyster adductor muscle, gill, and hemolymph by plating on a selective and differential medium. Finally, we evaluated oyster immune response and total weight changes to these different environmental challenges.

## Materials and methods

### *Vibrio parahaemolyticus* culture media and growth conditions

*Vibrio parahaemolyticus* strain EB101 (ATCC 17802) was cultured on nutrient agar (Difco) supplemented with 3% NaCl or in nutrient broth (Difco) supplemented with 3% NaCl, subsequently referred to as NA and NB, respectively. CHROMagar™ Vibrio plates were used to select for and differentiate *V. parahaemolyticus* from *Vibrio cholerae* and *Vibrio vulnificus*. All plates were incubated at 37°C (Thermo Scientific Hertherm IGS180) and broth cultures were shaken at 225 rpm and incubated at 37°C (Amerex Instruments SteadyShake 757 L). Tissues were homogenized in peptone water (Almuhaideb et al., 2020): 1% peptone (Difco) and 2% NaCl (Fisher Scientific).

### Verification of the absence of *Vibrio parahaemolyticus*, *Perkinsus marinus*, and *Haplosporidium nelsoni* in oysters

To verify the absence of both *V. parahaemolyticus* and other notable oyster pathogens that impact immune function (i.e., *Perkinsus marinus* and *Haplosporidium nelsoni*), a subset of oysters in culture conditions were evaluated before any experimentation began. To test for the presence of *V. parahaemolyticus*, oysters were sacrificed, and hemolymph from five oysters and hemolymph, adductor muscle, and gill tissues from four oysters were collected, homogenized, and centrifuged at 9,500 ×*g* for 15 s (Eppendorf centrifuge 5415D). The supernatant was then plated in duplicate on CHROMagar™ Vibrio plates, while hemolymph was directly plated in duplicate onto CHROMagar™ Vibrio. Levels of *P. marinus and H. nelsoni* (MDX disease) in hemolymph were investigated using methods from Gauthier and Fisher (1990) and Ford and Kanaley (1998).

### Oyster housing

All oysters were supplied by Copps Island Oysters, Norm Bloom & Son, located in Norwalk, CT. After oysters were transported to the University of Hartford, they were scrubbed of mud and epiphytes, notched, and housed in one of two 140-L tubs held at 20°C, and a salinity of 27‰. A water volume of ~80 L was recirculated and filtered using a Penn-Plax Cascade canister filter with a flow rate of 12 L/min. The chosen temperature and salinity parameters mimic the summer conditions of western Long Island Sound (NOAA, 2023). All water used in culture tubs and the exposure system was made using Instant Ocean™ sea salt that had been vigorously aerated for at least 96 h. Oysters were held in culture for at least 2 weeks before being placed in the experimental system. In both cultures and during experimentation, oysters were fed LBC Frozen Shellfish Diet from Reed Mariculture (0.25 g/oyster) three times per week. The presence of waste products (ammonia, nitrites, and nitrates) was tested at least once per week. Water changes were performed if levels of nitrogenous wastes (ammonia, nitrate, or nitrite) were found above 0.25 ppm. Levels in the exposure system did not have any detectable levels of waste products over either experimental exposure (data not shown). Supplementary Table S1 contains the recorded temperature (°C) and salinity (‰) values ± SD over the course of experimental exposures.

### Effect of salinity and temperature and *Vibrio parahaemolyticus* on oysters

To determine the interactive effects of elevated temperature, decreased salinity, and *V. parahaemolyticus* exposure on the immune response of *C. virginica*; 72 oysters were exposed to treatment conditions for a period of 28 days. Temperature and salinity levels were chosen based on the Intergovernmental Panel on Climate Change predictions for the year 2,300 (IPCC, 2014). Each treatment consisted of a sump where experimental conditions were monitored and maintained by probes connected to a Neptune APEX system. Treatment waters were pumped to three replicate tanks, randomly distributed on a shelving system. Effluent water was pumped back into the sump to be aerated and re-treated before returning to circulation (Supplementary Figure S1). Each line of the system housed approximately 60 L of water that was pumped into tanks at a flow rate of at least 1 L/min. Treatment lines consisted of a control, held at present-day temperature (20°C) and salinity (27‰) levels of western Long Island Sound (NOAA, 2023). The three remaining lines consisted of a single-stress treatment of decreased salinity (SS:DS; 20°C and 17‰), a single-stress treatment of elevated temperature (SS:ET; 27°C and 27‰), and a multistress treatment where both salinity and temperature were changed (MS: 27°C and 17‰). Each replicate tank housed six oysters (*n* = 18 per treatment). On day 0, each sump was inoculated with 10^4^ CFU/mL of *V. parahaemolyticus*.

### Bacterial load determined via viable plate counts on CHROMagar™ Vibrio media

At 7, 14, and 28 days of exposure, *n* = 2 oyster/tank (total of *n* = 6/treatment) were weighed (Ohaus Navigator Scale). Hemolymph was collected using a sterile 21-g needle and 3-mL syringe and was slowly added to a sterile 2-mL cryovial that was pre-cooled on ice to prevent clumping of hemocytes. After hemolymph collection, oysters were sacrificed, and gill and adductor muscle tissues were collected and placed in sterile 1.5-mL Eppendorf tubes over ice. Tissues were weighed and brought to 1 mL total volume with peptone water. The tissue samples were then ground using plastic pestles and spun at 9,500 ×g for 15 s. Tissue supernatant and hemolymph were plated onto CHROMagar™ Vibrio plates, and colonies were counted after approximately 24 h of incubation at 37°C.

### Immunology assays

After hemolymph collection (described above), the oyster immune response was quantified by total hemocyte counts (THC), phagocytic rate (PR; the number of particles engulfed by macrophages), and respiratory burst activity (RBA). Total hemocyte counts were conducted on vortexed hemolymph before 10 μL of the sample was loaded onto a hemocytometer. An Olympus CH-2 stereomicroscope was used to perform cell counts; the total count is expressed as cells/mL × 10^6^. Cells were counted by three individuals and averaged to eliminate bias in counting. The phagocytic rate of *C. virginica* hemocytes was quantified using the methods by Dang et al. (2011), modified for a 96-well plate. Briefly, a yeast suspension was prepared by mixing 2.5% *Saccharomyces cerevisiae* (i.e., Baker’s yeast) in 4% Congo Red in a 15 mM phosphate buffer saline (PBS) solution (137 mM NaCl, 2.7 mM KCl, 4.3 mM Na_2_HPO_4_, and 1.47 mM KH_2_PO_4_). This solution was autoclaved and used over the course of both experiments. Before each sampling day, an aliquot of the yeast cells was centrifuged at 1500 ×*g* for 10 min and washed three times with PBS (centrifuging at 3.5 ×*g* for 5 min after each wash). The cells were re-suspended in either 17‰ or 27‰ autoclaved seawater at 10^7^ cells/mL. On sampling days, 50 μL of hemolymph was added to microplate wells in duplicate; 50 μL of the yeast suspension (at either 17‰ or 27‰) was then added to each well and allowed to incubate for 30 min. After this incubation, the plate was centrifuged for 100 ×*g* in a Beckman Coulter Allegra X-30R tabletop centrifuge for 5 min. The hemolymph/yeast suspension was poured off, and each well rinsed three times with 15 mM PBS, centrifuging at 100 ×*g* for 5 min after each wash. Cells were fixed by incubating wells with 100 μL of a 50% EtOH solution with 1% acetic acid for 30 min. After which, the plate was read at 497 nm in a microplate reader (TECAN Infinite M Nano), with a standard curve made from the Congo red yeast suspension in PBS and PBS as a blank. Final phagocytic rates were expressed as yeast concentration phagocytized per milligram of hemolymph protein [determined using a Bradford Assay; Bradford (1976)]. Superoxide anion levels (i.e., respiratory burst activity; RBA) of oyster hemocytes were quantified by the reduction of nitroblue tetrazolium (NBT) as previously described (Dang et al., 2011). Final RBA numbers were standardized per milligram of hemolymph protein.

### Effect of multiple inoculations of *Vibrio parahaemolyticus* on oysters

As *V. parahaemolyticus* levels within the tanks declined over the first experimental exposure, a second, 28-day experiment was performed to discern whether multiple inoculations of bacteria would have a different impact on oyster survival and immune function. All treatment waters were generated using the multi-stress conditions outlined above (27°C and 17‰), which included the control; however, the control group was not inoculated with any bacteria. On day 0 of the experiment, *V. parahaemolyticus* was added at a concentration of 10^4^ CFU/mL to both the single-inoculation treatment (SI) and the multiple-inoculation treatment (MI). The MI group was inoculated once per week at a concentration of 10^4^ CFU/mL of *Vibrio* for the duration of the exposure. Bacterial load and metrics of oyster growth, hemolymph, and tissues were collected as described above.

### Bacterial molecular identification

Strains were streaked for isolation onto NA plates and incubated at 37°C overnight. One-quarter loopful (10 μL inoculating loops) of freshly streaked bacterial colonies were suspended in a 1.5-mL microcentrifuge tube containing 200 μL of 5% Chelex (BIO-RAD) in TE buffer. The tubes were vortexed so that no clumps were present and placed in a 100°C heating block for 10 min. Samples were vortexed for 30 s, placed on ice, and then subsequently centrifuged at 15,000 ×*g* for 10 min; 100 μL of the supernatant was transferred into a new 1.5-mL microcentrifuge tube. DNA concentrations and purity were assessed using a Nanodrop (DeNovix DS-11FX), and the samples were then subsequently stored at −20°C.

Each reaction mixture contained <250 ng of DNA, 1x GoTaq®polymerase (Promega), 0.4 μM of each primer (27F 5’ AGAGTTTGATCMTGGCTCAG-3′, and 1492R-5’ GGTTACCTTGTTACGACTT 3′) in a final volume of 25 μL. The amplification conditions were as follows: (i) 5 min at 95°C; (ii) 30 cycles of 30 s at 95°C, 30 s at 51°C, and 72°C for 1.5 min; and (iii) 5 min at 72°C. The PCR products were separated on a 0.8% agarose (American Bioanalytical) gel to verify amplification.

The PCR amplicons were purified using the QIAquick PCR Purification Kit (QIAGEN) according to the manufacturer’s instructions. The samples were stored at −20°C until used for sequencing. The 27F primer was used to obtain a partial sequence of the 16S rRNA gene. Sequences of at least 550 bp, which encompass the V1–V3 regions of the gene, were compared with the NCBI database using BLASTN (Altschul, 1990). Sanger sequencing reactions were completed at the W. M. KECK Biotechnology Resource Laboratory at the Yale University School of Medicine, New Haven, CT. Reactions contained 500 ng of template, 2 μL of 4 mM primer, in a total volume of 18 μL.

### Statistical analysis

Prism 7.0 was used to perform all statistical analysis. To determine whether the number of bacteria changed between environmental conditions within a particular tissue at a given time point, a two-way ANOVA was performed. Tests for normality (Kolmogorov–Smirnov test) failed for these datasets; therefore, a two-way ANOVA on rank-transformed data was used to test for significant differences. Tukey’s method was used for *post-hoc* multiple comparisons between the different conditions (Macey et al., 2008; Rayner and Best, 2013). The log-rank test was used to determine differences in the survival curves between the different treatment groups. Oyster weight and immunology data were analyzed by a two-way ANOVA for the main effects of time, treatment, and interaction between these main effects. Levene’s test for homogeneity of variance was used, along with a Shapiro–Wilk test for normal distribution of data. Tukey’s HSD *post hoc* test were performed to investigate the differences between treatments at each time point of exposure. Findings were considered significant at *p* < 0.05.

## Results

### Absence of culturable bacteria and the protozoa, *Perkinsus marinus* and *Haplosporidium nelsoni*, in tissue

To determine the potential impact of decreased salinity and elevated temperature would have on the ability of eastern oysters to resist colonization by *V. parahaemolyticus*, we first verified the absence of *V. parahaemolyticus* from oysters collected from growing beds in western Long Island Sound. Prior to experimentation, we were unable to culture *V. parahaemolyticus* or any other organisms when homogenized oyster tissue or hemolymph was plated from nine oysters on CHROMagar™ Vibrio plates (data not shown). *Perkinsus marinus* and *Haplosporidium nelsoni* were also not detected (data not shown).

### Effect of salinity, temperature, and *Vibrio parahaemolyticus* on oyster microbiota

**Figure 1:**
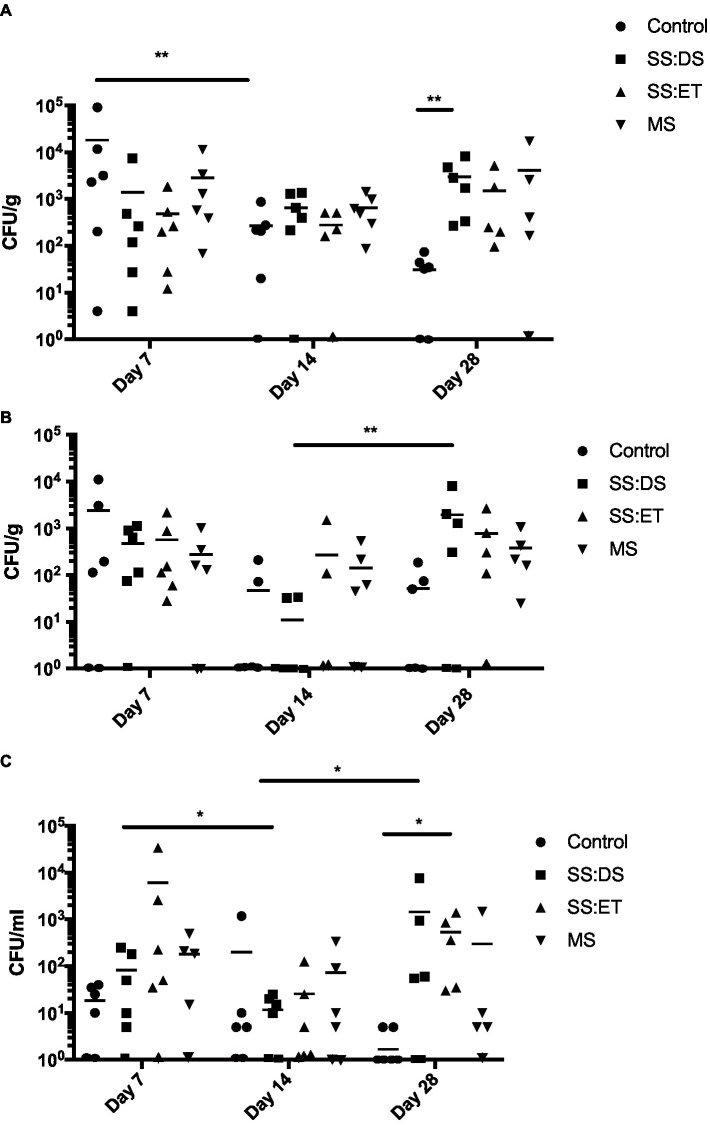
Total culturable bacteria recovered from oyster tissue. Bacterial numbers in adductor muscle **(A)**, gill **(B)**, and hemolymph **(C)** at days 7, 14, and 28 post-tank inoculation with 10^4^ CFU/mL of *Vibrio parahaemolyticus* in each of the treatment groups, which consisted of the control, single stress: decreased saline (SS:DS), single stress: elevated temperature (SS:ET), and multistress: decreased saline and increased temperature (MS). Significant differences in bacterial burdens between treatment groups were assessed by analyzing rank-transformed culturable bacterial data by two-way ANOVA using Tukey’s post-test for multiple comparisons and are indicated by asterisks (^*^p < 0.05, ^**^p < 0.01). Each data point represents results from one oyster. Data points located on the horizontal axis indicate values were below the limit of detection. Horizontal lines represent mean values of six oysters at days 7 and 14, and 5–6 oysters at day 28.

We observed bacterial growth in all tissues, conditions, and time points (Figures 1A–C), where total bacterial numbers ranged from a mean of 1.6 CFU/mL in control conditions in hemolymph at day 28 (Figure 1C) to a mean of 1.8 × 10^4^ CFU/g in control conditions in adductor muscle at day 7 (Figure 1A). Total bacterial levels remained elevated from day 7 to day 28 under SS:DS, SS:ET, and MS treatments in all tissues (Figure 1), while total bacterial numbers in the control group appeared to decrease over time, with a statistically significant decrease in numbers (Tukey’s, *p* < 0.01) in adductor muscle from days 7 to 28 (Figure 1A).

**Figure 2:**
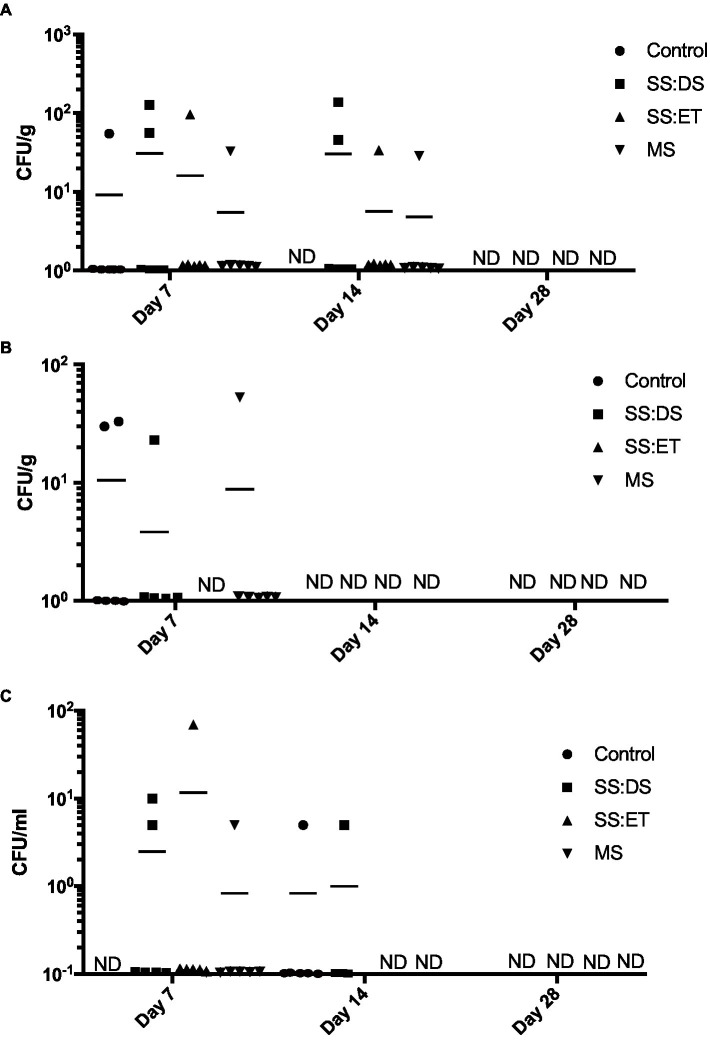
*Vibrio parahaemolyticus* recovered from oyster tissue. *Vibrio parahaemolyticus* numbers in adductor muscle **(A)**, gill **(B)**, and hemolymph **(C)** at days 7, 14, and 28 post-tank inoculation with 10^4^ CFU/mL of *V. parahaemolyticus* in each of the treatment groups, which consisted of the control, single stress: decreased saline (SS:DS), single stress: elevated temperature (SS:ET), and multistress: decreased saline and increased temperature (MS). Significant differences in bacterial burdens between treatment groups were assessed by analyzing rank-transformed culturable bacterial data by two-way ANOVA using Tukey’s post-test for multiple comparisons. Each data point represents results from one oyster. Data points located on the horizontal axis indicate values were below the limit of detection. Horizontal lines represent mean values of six oysters at days 7 and 14, and five to six oysters at day 28. ND, not detected.

Then, we determined the levels of *V. parahaemolyticus* by counting mauve colonies on the CHROMagar™ Vibrio plates. At day 7, we did not detect *V. parahaemolyticus* in more than two oyster adductor muscles (Figure 2A), gill (Figure 2B), and hemolymph from a particular treatment (Figure 2C). At day 14, *V. parahaemolyticus* was only detectable in adductor muscle under SS:DS (two oysters), SS:ET (one oyster), and MS (one oyster) conditions (Figure 2A) and hemolymph under control (one oyster) and SS:DS oyster conditions (one oyster) (Figure 2C). *V. parahaemolyticus* was completely cleared from oyster gill by day 14 under all conditions (Figure 2B) and from adductor muscle, gill, and hemolymph under all conditions by day 28 (Figure 2).

**Figure 3:**
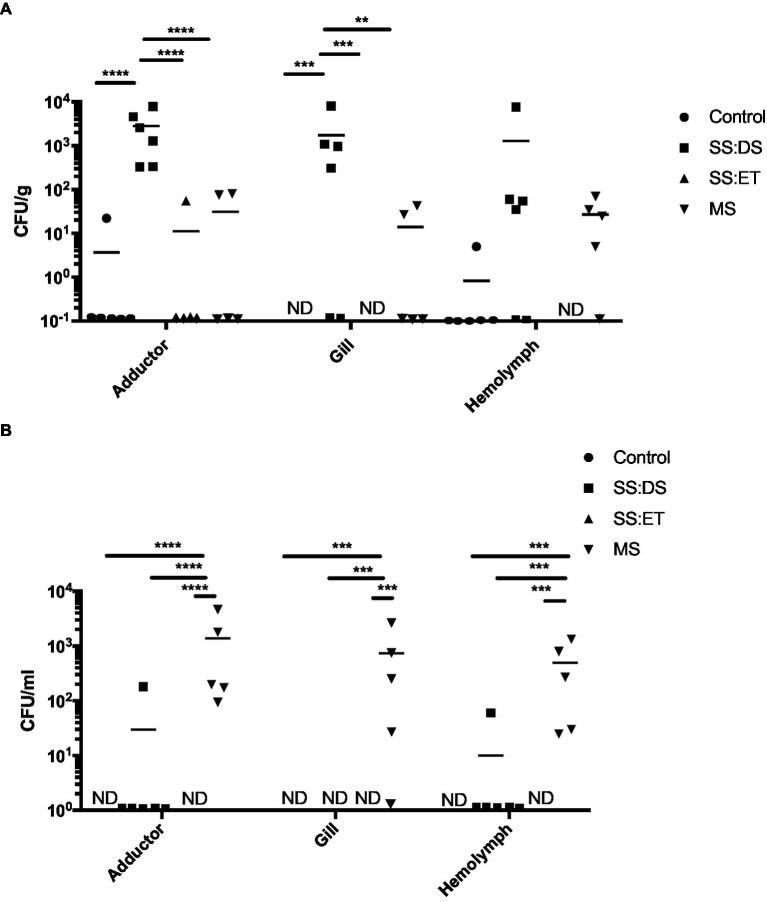
Non-*Vibrio parahaemolyticus Vibrio* species recovered from oyster tissue. Bacterial numbers of *Vibrio aestuarianus* **(A)** and *Vibrio harveyi* **(B)** in adductor muscle, gill, and hemolymph at day 28 post-tank inoculation with 10^4^ CFU/mL of *V. parahaemolyticus* in each of the treatment groups, which consisted of the control, single stress: decreased saline (SS:DS), single stress: elevated temperature (SS:ET), and multistress: decreased saline and increased temperature (MS). Significant differences in bacterial burdens between treatment groups were assessed by analyzing rank-transformed culturable bacterial data by two-way ANOVA using Tukey’s post-test for multiple comparisons and are indicated by asterisks (^*^p < 0.05, ^**^p < 0.01, ^***^p < 0.001, ^****^p < 0.0001). Each data point represents results from one oyster. Data points located on the horizontal axis indicate values were below the limit of detection. Horizontal lines represent mean values of six oysters at days 7 and 14, and five to six oysters at day 28. ND, not detected.

In addition to *V. parahaemolyticus*, 13 different types of colonies were observed on the CHROMagar™ Vibrio plates throughout the duration of the experiment, and their identities were determined via 16S rRNA gene sequencing (Supplementary Table S2). On day 28, we noticed pronounced variations in colony numbers from two different types of bacteria, which were identified as *Vibrio aestuarianus* and *Vibrio harveyi*. *Vibrio aestuarianus* numbers were significantly higher in the SS:DS treatment group in adductor muscle and gill when compared to the control (Tukey’s, *p* < 0.0001; *p* < 0.001), SS:ET (Tukey’s, *p* < 0.0001; *p* < 0.001), and MS (Tukey’s, *p* < 0.0001; *p* < 0.01; Figure 3A). While *V. aestuarianus* numbers in hemolymph in the SS:DS treatment group were elevated when compared to the control and SS:ET, they were not found to be statistically different (*p* = 0.055, *p* = 0.063, respectively; Figure 3A). This point requires further investigation, and a higher n-value might result in significance in this tissue. Mean *V. aestuarianus* numbers were greater than 10^3^ CFU/g or mL in all three tissues (Figure 3A). *Vibrio harveyi* numbers were significantly higher in the MS treatment group in adductor muscle, gill, and hemolymph when compared to the control (Tukey’s, *p* < 0.0001 in all cases). SS:DS (Tukey’s, *p* < 0.001 in all cases), and SS:ET (Tukey’s, *p* < 0.001 in all cases) treatment groups (Figure 3B). Mean *V. harveyi* numbers in the MS treatment ranged from 4.0 × 10^2^ CFU/mL in the hemolymph to 1.4 × 10^3^ CFU/g in adductor muscle, while it was not detected in any of the tissues from the control and SS:ET treatments, and SS:DS treatment from gill (Figure 3B).

### Impact of multiple *Vibrio parahaemolyticus* inoculations on oyster microbiota

**Figure 4:**
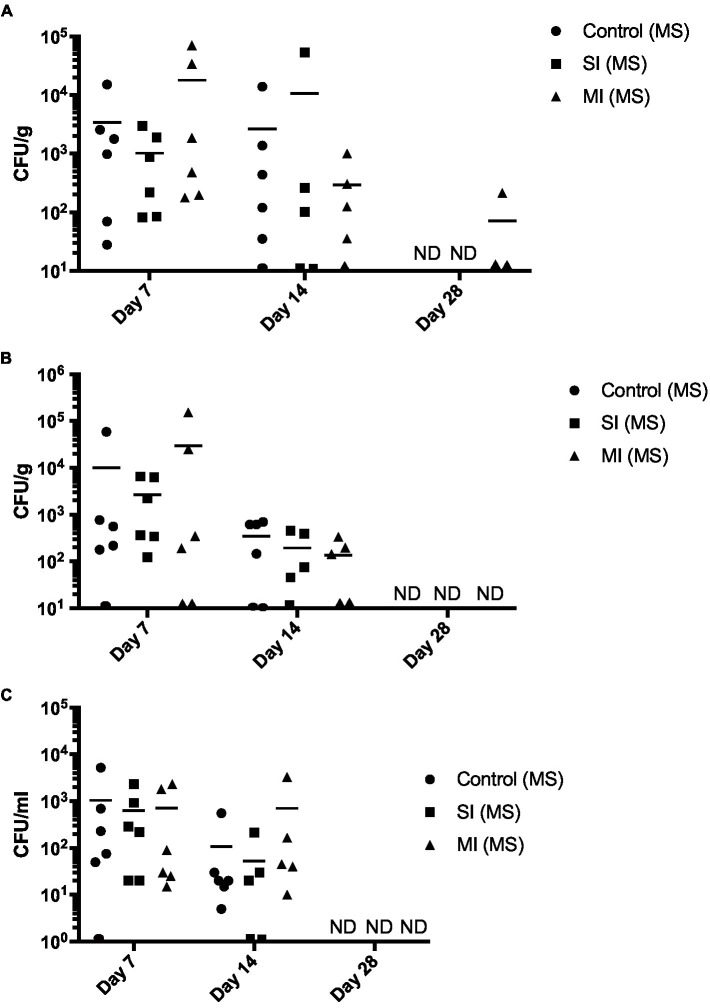
Total culturable bacteria recovered from oyster tissue. Bacterial numbers in adductor muscle **(A)**, gill **(B)**, and hemolymph **(C)** at days 7, 14, and 28 post-tank inoculation in each of the treatment groups, which consisted of the control (MS) (multistress: decreased saline and increased temperature), multistress: single inoculation (MS: SI) with 10^4^ CFU/mL of *V. parahaemolyticus*, and multistress: multi-inoculation (MS:MI) with 10^4^ CFU/mL of *V. parahaemolyticus* every 7 days for the duration of the experiment. Significant differences in bacterial burdens between treatment groups were assessed by analyzing rank-transformed culturable bacterial data by two-way ANOVA using Tukey’s post-test for multiple comparisons. Each data point represents results from one oyster. Data points located on the horizontal axis indicate values were below the limit of detection. Horizontal lines represent mean values of six oysters at day 7, ± SEM of five to six oysters at day 14, and ± SEM of one to five oysters on day 28. ND, not detected.

As we were unable to detect *V. parahaemolyticus* in the tanks after 5 days post-inoculation (Supplementary Figure S2), we decided to include a multi-inoculation treatment group in the subsequent experiment (Supplementary Figure S3). Culturable bacteria were detected at days 7 and 14 in all tissue types and all treatment conditions (Control, SI, and MI) (Figure 4). Total mean bacterial numbers ranged from 50 CFU/mL at day 14 in the hemolymph from the SI treatment (Figure 4C) to over 10^4^ CFU/g at day 7 in the gill from the MI treatment (Figure 4B). By day 28, bacteria were not detected in gill or hemolymph from any of the treatment conditions (Figures 4B,C), and only the adductor muscle from the MI group had detectable levels of culturable bacteria (Figure 4A).

**Figure 5:**
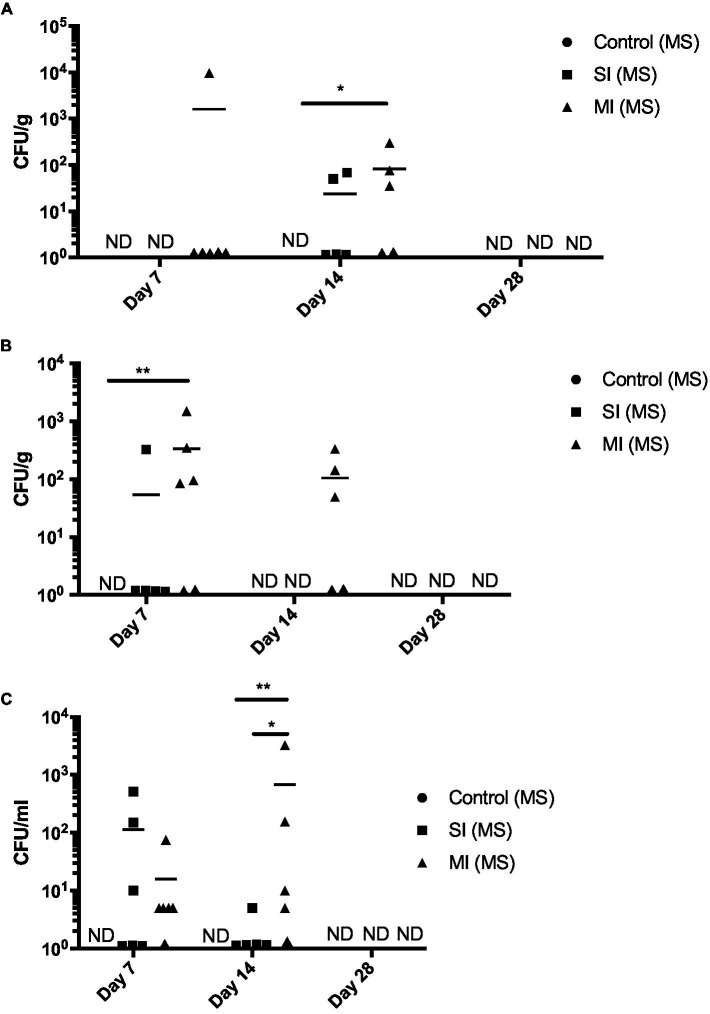
*Vibrio parahaemolyticus* recovered from oyster tissue. *Vibrio parahaemolyticus* numbers in adductor muscle **(A)**, gill **(B)**, and hemolymph **(C)** at days 7, 14, and 28 post-tank inoculation with 10^4^ CFU/mL of *V. parahaemolyticus* in each of the treatment groups, which consisted of the control (MS) (multistress: decreased saline and increased temperature), multistress: single inoculation (MS: SI) with 10^4^ CFU/mL of *V. parahaemolyticus*, and multistress: multi-inoculation (MS:MI) with 10^4^ CFU/mL of *V. parahaemolyticus* every 7 days for the duration of the experiment. Significant differences in bacterial burdens between treatment groups were assessed by analyzing rank-transformed culturable bacterial data by two-way ANOVA using Tukey’s post-test for multiple comparisons and are indicated by asterisks (^*^p < 0.05, ^**^p < 0.01). Each data point represents results from one oyster. Data points located on the horizontal axis indicate values below the limit of detection. Horizontal lines represent mean values of six oysters at day 7, ± SEM of five to six oysters at day 14, and ±SEM of one to five oysters at day 28. ND, not detected.

Then, we determined the amount of *V. parahaemolyticus* in oyster tissues by examining the CHROMagar™ Vibrio plates for mauve colonies. Throughout the duration of the experiment (day 7–28), *V. parahaemolyticus* was not detected in the control in any of the tissues (Figure 5). Oysters were able to clear *V. parahaemolyticus* by day 28 in all tissues and treatments (Figure 5). Mean *V. parahaemolyticus* values were higher in the multi-inoculation treatment when compared to the single-inoculation treatment group at days 7 and 14 in adductor muscle (Figure 5A) and gill (Figure 5B) and higher (Tukey’s, *p* < 0.05) at day 14 in hemolymph (Figure 5C). We observed elevated *V. harveyi* numbers at day 7 (Supplementary Figure S4), which were consistent with those observed in the first trial at day 28 in the MS treatment. However, we did not detect *V. harveyi* in any of the oyster tissues at day 14 or 28 (data not shown). We also detected low numbers of *V. aestuarianus* in all three treatments at day 7 and in the control and MI(MS) treatments at day 14, but like *V. harveyi*, it was cleared by day 28 (data not shown).

### Effect of salinity, temperature, and *Vibrio parahaemolyticus* on oyster immune response

**Figure 6:**
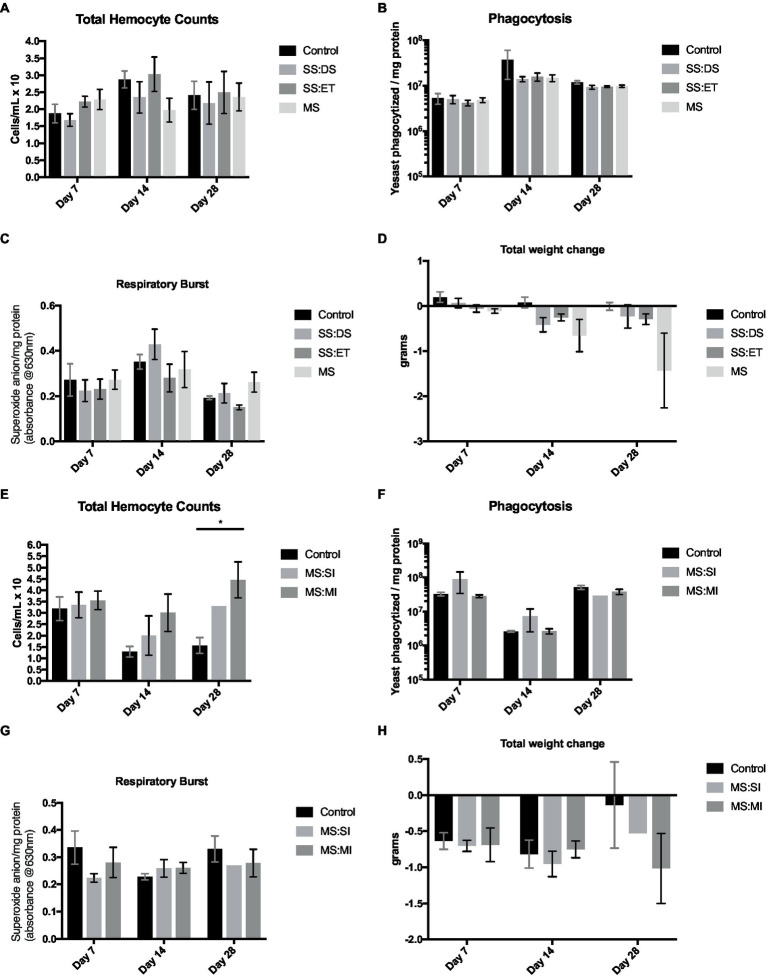
Measurements of total hemocyte counts (THC; cells/mL × 10^6^ **A,E**) ± SE, phagocytosis (yeast phagocytized/mg protein; **B,F**) ± SE, respiratory burst activity (RBA; absorbance at 630 nm/mg protein; **C,G**) ± SE, and total weight change (g; **D,H**) ± SE of *n* = 6 oyster at 7, 14 and 28 days of acclimation. Oysters were exposed to either control conditions (black bars), a single stress of decreased salinity (SS:DS; medium gray; **A–D**), a single stress of elevated temperature (SS:ET; dark gray; **A–D**), and a multistress treatment (MS; light gray; **A–D**). Oyster measurements in **E–H** were exposed to either a control treatment (black bars), or multistress conditions with either a single inoculation of *V. parahaemolyticus* (MS:SI; light gray bars), or multiple inoculations of bacteria (MS:MI; dark gray). Asterisks indicate a significant difference from control conditions (*p* < 0.05).

Oyster weight significantly declined over the course of the first 28-day exposure (*F*_2,57_ = 3.902, *p* = 0.026); two-way ANOVA showed there was a significant effect of treatment (*F*_3,57_ = 5.173, *p* = 0.003), with oysters exposed to the combination of elevated temperature and decreased salinity showing the greatest decline in mass (Figure 6D). Oyster weights in the multistress treatment were significantly different from control values at both 14 and 28 days of exposure (Tukey’s, *p* = 0.003 in both cases; Figure 6D). While all oysters decreased in mass over 28 days in the second experimental exposure, multiple inoculations of *V. parahaemolyticus* did not negatively impact oysters more than a single inoculation (Figures 6H). There were no statistical differences seen in the response of oysters to multiple stressors in the initial 28-day exposure and the second exposure (*p* = 0.52); comparing the weight loss of oysters in the control of the first exposure to the single- and multiple-inoculation oysters in the second showed significant change (Tukey’s, *p* = 0.019and *p* = 0.001, respectively).

Small changes in hemocyte numbers across treatments and a significant main effect of time were noted (*F*_2,51_ = 3.381, *p* = 0.042; Figure 6A). However, there was no significant effect of treatment; the two-way ANOVA from the second inoculation experiment showed significant main effects of both acclimation time (*F*_2,31_ = 3.543, *p* = 0.041) and treatment (*F*_2,31_ = 5.287, *p* = 0.011). The hemocyte counts were significantly elevated in the multiple-inoculation oysters vs. control oysters at 28 days of exposure (Tukey’s, *p* = 0.003; Figure 6E).

Respiratory burst activity showed significant changes over time (*F*_2,54_ = 7.038, *p* = 0.002), with levels increasing at 14 days of exposure and then declining by day 28 (Figure 6C). Oyster in the second exposure did not display any notable changes in measurements of this metric (Figure 6G).

In our initial exposure, phagocytosis levels were significantly different over time (*F*_2,57_ = 4.939, *p* = 0.011) with 14-day phagocytosis rates elevated above 7- and 28-day values (Tukey’s, *p* = 0.003 and 0.048, respectively; Figure 6B). At this time point, there was a significant difference found between control levels, and levels of phagocytosis in the single stress of decreased salinity (Tukey’s, *p* = 0.024), elevated temperature (Tukey’s, *p* = 0.037), and the multistress treatment (Tukey’s, *p* = 0.030). Multiple inoculations of *Vibrio* did not influence the phagocytosis rates in oysters (Figure 6F).

**Figure 7:**
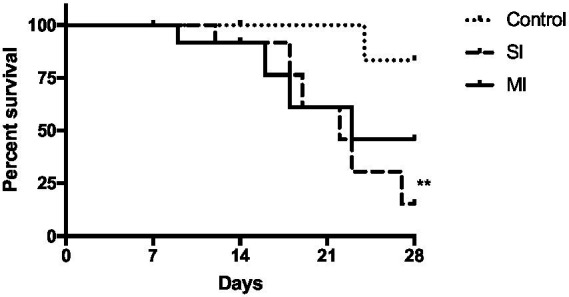
Survival curve of oysters from control (MS), single inoculation (MS:SI), and multi-inoculation (MS:MI) over the 28-day experiment. Significant differences in survival curves between the groups were assessed using the log-rank test and are indicated by asterisks (^**^p < 0.01).

Over the duration of the second experiment, we noted a significant increase in mortality with the single (Tukey’s, *p* < 0.01) inoculation compared to oysters in the control group (Figure 7). Although not significant, it also appears that the multiple-inoculation group is trending toward higher mortality (Tukey’s, *p* = 0.1) when compared to oysters in the control group (Figure 7).

## Discussion

In the current study, we assessed the ability of *C. virginica* to resist *V. parahaemolyticus* infection under climate change conditions. To our knowledge, this is the first study that examined the impact of elevated temperature and decreased salinity in the presence of *V. parahaemolyticus* on oysters. Studies have shown that temperature has a major impact on the relationship between *V. parahaemolyticus* and oysters. For example, as temperature rises, *V. parahaemolyticus* numbers have been shown to increase within seawater and oyster, which has consequently led to an increase in human infections (Iwamoto et al., 2010; Almuhaideb et al., 2020). In addition to environmental factors (e.g., temperature and salinity) influencing the number of microorganisms within seawater, they can also impact oyster health, thereby synergistically increasing the likelihood of microbial infection (Hegaret et al., 2004; Rybovich et al., 2016; Du et al., 2021; Stevick et al., 2021). In the face of global climate change, these numbers are expected to rise (Patz et al., 2005; Harvell et al., 2009).

Most animals are colonized by a complex community of microorganisms, which have an essential role in maintaining host health. In humans, for example, several factors, such as antibiotic usage, poor diet, or a weakened immune system, can lead to an imbalance of the normal microbial flora causing the proliferation of opportunistic pathogens, which can lead to a disease state. Numerous studies have revealed that environmental perturbations can impact the native bacterial community within oyster tissue. For example, temperature (Lokmer et al., 2016; Green et al., 2018), low salinity (Li et al., 2022), exogenously added bacteria (Froelich B and Oliver JD, 2013), and viral infection (Petton et al., 2015; de Lorgeril et al., 2018) have all been shown to alter the oyster microbiome and in most cases lead to an increase in oyster mortality. Our results are consistent with these studies, as we demonstrated the presence of *V. parahaemolyticus* or the combination of elevated temperature and decreased salinity led to the proliferation of endogenous bacteria within oyster adductor muscle, gill, and hemolymph. It is possible that these environmental stressors negatively impacted the oyster immune system, which keeps the native microbial flora in check and below the limit of our detection. Previous studies exploring changes in temperature, salinity, or the combination of these environmental stressors and the immune response of bivalves have produced varying results. *Crassostrea virginica* exposed to an increase in temperature from 20 to 28°C for 1 week led to a decrease in phagocytosis in two different experiments (Hegaret et al., 2003, 2004), yet a 20-day exposure that increased temperature from 10 to 25°C resulted in an increase in phagocytosis in the same species (Chu and La Peyre, 1993). A 14-day exposure to a temperature elevation of 5°C from control significantly increased phagocytosis and total hemocyte counts in both *Crassostrea gigas* and *Mytilus galloprovincialis* (Rahman et al., 2019). A 6-month exposure of *Mytilus edulis* to temperatures 4°C above ambient levels increased all immunological parameters used in this study (total hemocyte counts, phagocytosis, and respiratory burst activity; Mackenzie et al., 2014). Taken together, these studies demonstrate that the bivalve immune response takes at least 2 weeks to respond to an elevation in temperature, which was also noted in this study. Osmotic stress responses seem to be more consistent, with hypo-osmotic exposure leading to a more intense inflammatory response, increases in total hemocyte counts, and respiratory burst activity (Fisher and Newell, 1986; Matozzo et al., 2007; Li et al., 2022; Pérez-Velasco et al., 2022). When combined, the interactive effects of temperature and salinity appear to enhance the immune response in rock oysters, *Saccostrea glomerata* over a 1-week exposure, resulting in an increase in phagocytosis (Ertl et al., 2019). Exposure to elevated temperature and decreased salinity for 7 days also increased phagocytosis as well as total hemocyte counts in clam, *Ruditapes philippinarum* (Munari et al., 2011). As stated above, many changes in immunological parameters in this study were seen at 14 days of exposure. However, total hemocyte counts in the second exposure were significantly increased in oysters that were inoculated with *V. parahaemolyticus* at 28 days. The 14-day hemocyte counts were elevated above control values, but this was not statistically significant. It is possible a higher number of oysters would illustrate a more definitive result.

In our first experimental trial, we examined the ability of oysters to resist *V. parahaemolyticus* infection under elevated temperature (SS:ET), decreased salinity (SS:DS), and both elevated temperature and decreased salinity (MS). We detected *V. parahaemolyticus* among the total culturable bacteria we recovered from oyster post-challenge. Interestingly, despite *V. parahaemolyticus* being detected in most tissues and treatment groups at day 7 post-challenge, it was undetectable by day 28. We hypothesize two reasons for this finding. First, it is possible that the oyster immune response adapted to the environmental stress over the 4-week trial and eliminated or reduced *V. parahaemolyticus* to below detectable levels. A second possibility is that the native oyster microbiota can protect against and/or potentially outcompete exogenous pathogens, which has been demonstrated in previous studies (Lokmer and Mathias Wegner, 2015; de Lorgeril et al., 2018). Research has shown that bacteria are quickly taken up by bivalves, but bacterial levels are then rapidly reduced after a few days or a week. This depuration of exogenous bacteria has largely been attributed to the presence of the native microbial flora, which outcompete the exogenous bacteria for available surface area (Froelich and Noble, 2014). This line of reasoning tracks with the overall lack of immune response noted in this study, and the decline of oyster mass can therefore be attributed to impacts of elevated temperature and decreased salinity.

Among the organisms that we were able to detect and significantly proliferated after environmental challenge in the first experimental exposure (and to a lesser extent in exposure 2) were *Vibrio aestuarianus* and *Vibrio harveyi*, in the SS:DS and MS groups, respectively. These two organisms comprise part of the oyster microbiome, albeit at low levels and potentially in an inactive state (Froelich B and Oliver J, 2013; Green et al., 2018). Interestingly, both organisms are also considered oyster pathogens and have previously been shown to contribute to oyster mortality (Garnier et al., 2007; Travers et al., 2015; Green et al., 2018; King et al., 2019). Previously, it has been shown that heat stress on oysters led to an increase in the abundance of total bacteria, which included *V. harveyi.* The elevated bacterial numbers were then directly linked to oyster mortality (Green et al., 2018). Taken together, the environmental stressors (i.e., *V. parahaemolyticus*, decreased salinity, and elevated temperature) examined in this study inhibited host physiology and allowed for the proliferation of the native flora, some of which are opportunistic pathogens of the genus *Vibrio*.

Our results from the second experiment suggest that elevated temperature and decreased salinity impact bacterial numbers, regardless of the presence or absence of *V. parahaemolyticus*. Additionally, we observed higher oyster mortality in the groups treated with *V. parahaemolyticus* than the (MS) control. It is probable that the microbial composition and total numbers of bacteria are indeed different between these treatments, but it is beyond the scope of our detection method to confirm this. We had several oysters die prior to day 28, in both the SI and MI groups. Therefore, it is intriguing to speculate that oysters were either able to reduce native bacterial numbers to pre-treatment levels or die. This is consistent with previous findings that linked environmental stress to the proliferation of native flora, which resulted in oyster mortality (Petton et al., 2015; Richards et al., 2015).

To that end, it was surprising that oysters in the MS (SI) group in trial two experienced significant mortality, while the MS group in the first experimental exposure, which was also inoculated with *V. parahaemolyticus*, did not. A possible explanation for this discrepancy is that it has been previously suggested that the microbiomes of healthy oysters are more diverse and contribute to their resistance to environmental stressors (Stevick et al., 2021). Interestingly, we detected 13 different organisms from oysters in the first experiment compared to two in the second. Therefore, perhaps the lack of microbial diversity within the oysters in the second exposure made them more susceptible to *V. parahaemolyticus* and the multistress condition of elevated temperature and decreased salinity.

Various environmental stresses brought on by climate change can impact oyster physiology as well as their microbiome. This has repercussions for oyster, human, and environmental health. As bacterial numbers increase, some of which are known oyster and human pathogens, it leads to an increase in oyster mortality, which subsequently impacts the aquatic ecosystem as oysters are an important keystone species (Kennedy, 1996). In addition, oysters are frequently consumed raw by humans, which poses a risk for food-borne illness. Climate change impacts ecosystems in various ways (i.e., via increased temperature, decreased salinity, ocean acidification, and decreased dissolved oxygen). This study is a first step in understanding the synergistic effects of two of these environmental stressors brought on by climate change in the presence of a pathogenic marine bacterium. Future studies will need to examine the full scope of climate change on its impact on oyster health. A culture-independent approach to examining the effect these stressors have on oyster microbiota will provide a more thorough picture of the extent to which these changes impact the native oyster flora. Furthermore, understanding how the environmental alterations brought about by climate change affect estuarine environments will be paramount in fully understanding the health risks brought on by climate change.

## Data availability statement

The sequencing data is available via GenBank Accession Numbers PP379220-PP379232.

## Ethics statement

The manuscript presents research on animals that do not require ethical approval for their study.

## Author contributions

OR: Formal analysis, Investigation, Writing – original draft, Writing – review & editing. SI: Investigation, Writing – review & editing. RL: Investigation, Writing – review & editing. TM: Investigation, Writing – review & editing. LE: Conceptualization, Formal analysis, Funding acquisition, Investigation, Methodology, Project administration, Resources, Supervision, Writing – original draft, Writing – review & editing. AS: Conceptualization, Formal analysis, Funding acquisition, Investigation, Project administration, Resources, Supervision, Writing – original draft, Writing – review & editing.

## Supplementary material

SUPPLEMENTARY FIGURE S1 Diagram of the experimental system setup.

SUPPLEMENTARY FIGURE S2 *Vibrio parahaemolyticus* levels in oyster tanks during experimental trial 1. Approximately 10^4^ CFU/ml of *V. parahaemolyticus* was inoculated in each of the treatment groups, which consisted of the control, single stress: decreased saline (SS:DS), single stress: elevated temperature (SS:ET), and multi stress: decreased saline and increased temperature (MS). *Vibrio parahaemolyticus* levels were undetectable in each of the groups after 120 h.

SUPPLEMENTARY FIGURE S3 *Vibrio parahaemolyticus* levels in oyster tanks during experimental trial 2. Approximately 10^4^ CFU/ml of *V. parahaemolyticus* was inoculated in each of the treatment groups, which consisted of the control (MS) (multi stress: decreased saline and increased temperature), multi stress: single inoculation (MS:SI) with 10^4^ CFU/ml of *V. parahaemolyticus*, and multi stress: multi-inoculation (MS:MI) with 10^4^ CFU/ml of *V. parahaemolyticus* every 7 days for the duration of the experiment. *Vibrio parahaemolyticus* levels were undetectable in the control group for the duration of the experiment and after 7 days in the MS:SI treatment.

SUPPLEMENTARY FIGURE S4 *Vibrio harveyi* recovered from oyster tissue. Bacterial numbers of *Vibrio harveyi* in adductor muscle, gill, and hemolymph at day 7 post-tank inoculation with 10^4^ CFU/ml of *V. parahaemolyticus* in each of the treatment groups, which consisted of the control (MS) (multi stress: decreased saline and increased temperature), multi stress: single inoculation (MS:SI) with 10^4^ CFU/ml of *V. parahaemolyticus*, and multi stress: multi inoculation (MS:MI) with 10^4^ CFU/ml of *V. parahaemolyticus* every 7 days for the duration of the experiment. No significant differences of bacterial burdens were observed between treatment groups. Each data point represents results from one oyster. Data points located on the horizontal axis indicate values were below the limit of detection. Horizontal lines represent mean values.
