# Supplementary material for: Elevated temperature and decreased salinity impacts on exogenous Vibrio parahaemolyticus infection of eastern oyster, Crassostrea virginica
Source: Front Microbiol. 2024 Jul 4;15:1388511. doi: 10.3389/fmicb.2024.1388511 (PMC11257037; doi:10.3389/fmicb.2024.1388511)
Supplement: SUPPLEMENTARY FIGURE S1 — Diagram of the experimental system setup. [file Data_Sheet_1.zip › Table S1.docx]

| **Control Temperature (°C)** | **Control Salinity (‰)** | **SS:DS Temperature (°C)** | **SS:DS Salinity (‰)** | **SS:ET Temperature (°C)** | **SS:ET Salinity (‰)** | **MS Temperature (°C)** | **MS Salinity (‰)** |
| --- | --- | --- | --- | --- | --- | --- | --- |
| 20.0 ± 0.78 | 23.1 ± 1.8 | 20.0 ± 0.4 | 17.2 ± 0.7 | 26.5 ± 0.4 | 26.9 ± 0.4 | 26.6 ± 0.5 | 16.9 ± 0.5 |
| **Control Temperature (°C)** | **Control Salinity (‰)** | **MS:SI Temperature (°C)** | **MS:SI Salinity (‰)** | **MS:MI Temperature (°C)** | **MS:MI Salinity (‰)** |  |  |
| 27.4 ± 0.3 | 16.8 ± 0.3 | 27.2 ± 0.3 | 16.7 ± 0.4 | 27.2 ± 0.3 | 16.8 ± 0.5 |  |  |
