# Supplementary material for: Elevated temperature and decreased salinity impacts on exogenous Vibrio parahaemolyticus infection of eastern oyster, Crassostrea virginica
Source: Front Microbiol. 2024 Jul 4;15:1388511. doi: 10.3389/fmicb.2024.1388511 (PMC11257037; doi:10.3389/fmicb.2024.1388511)
Supplement: SUPPLEMENTARY FIGURE S1 — Diagram of the experimental system setup. [file Data_Sheet_1.zip › Table S2.docx]

Table S2. Organisms identified via 16S rRNA gene sequencing.

|  |
| --- |
| *Shewanella algae* |
| *Shewanella aquimarina* |
| *Vibrio aestuarianus* |
| *Vibrio brasiliensis* |
| *Vibrio campbellii* |
| *Vibrio fortis* |
| *Vibrio harveyi* |
| *Vibrio jasicida* |
| *Vibrio mediterraneii* |
| *Vibrio natriegens* |
| *Vibrio owensii* |
| *Vibrio rotiferianus* |
| *Vibrio sinaloensis* |
|  |
