# Supplementary figures and images for: Elevated temperature and decreased salinity impacts on exogenous Vibrio parahaemolyticus infection of eastern oyster, Crassostrea virginica
Source: Front Microbiol. 2024 Jul 4;15:1388511. doi: 10.3389/fmicb.2024.1388511 (PMC11257037; doi:10.3389/fmicb.2024.1388511)

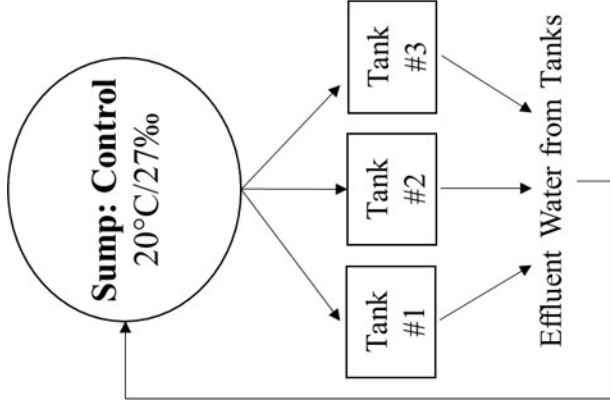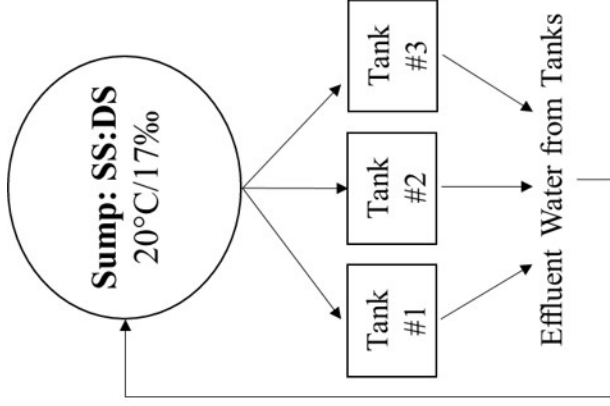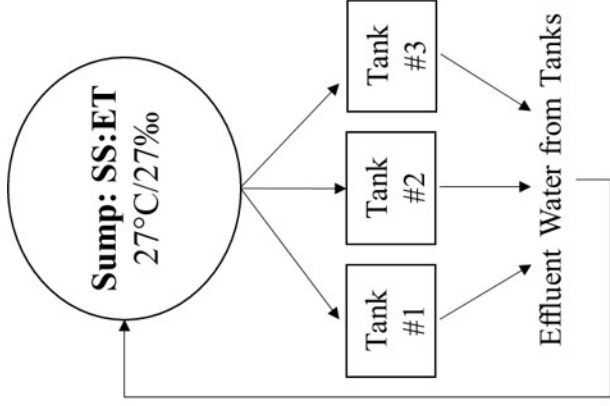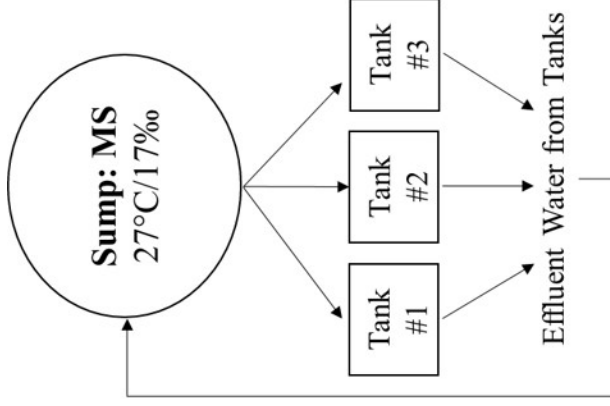

Supplement: SUPPLEMENTARY FIGURE S1 — Diagram of the experimental system setup. [file Data_Sheet_1.zip › FigS1.pdf]

Fig. S2

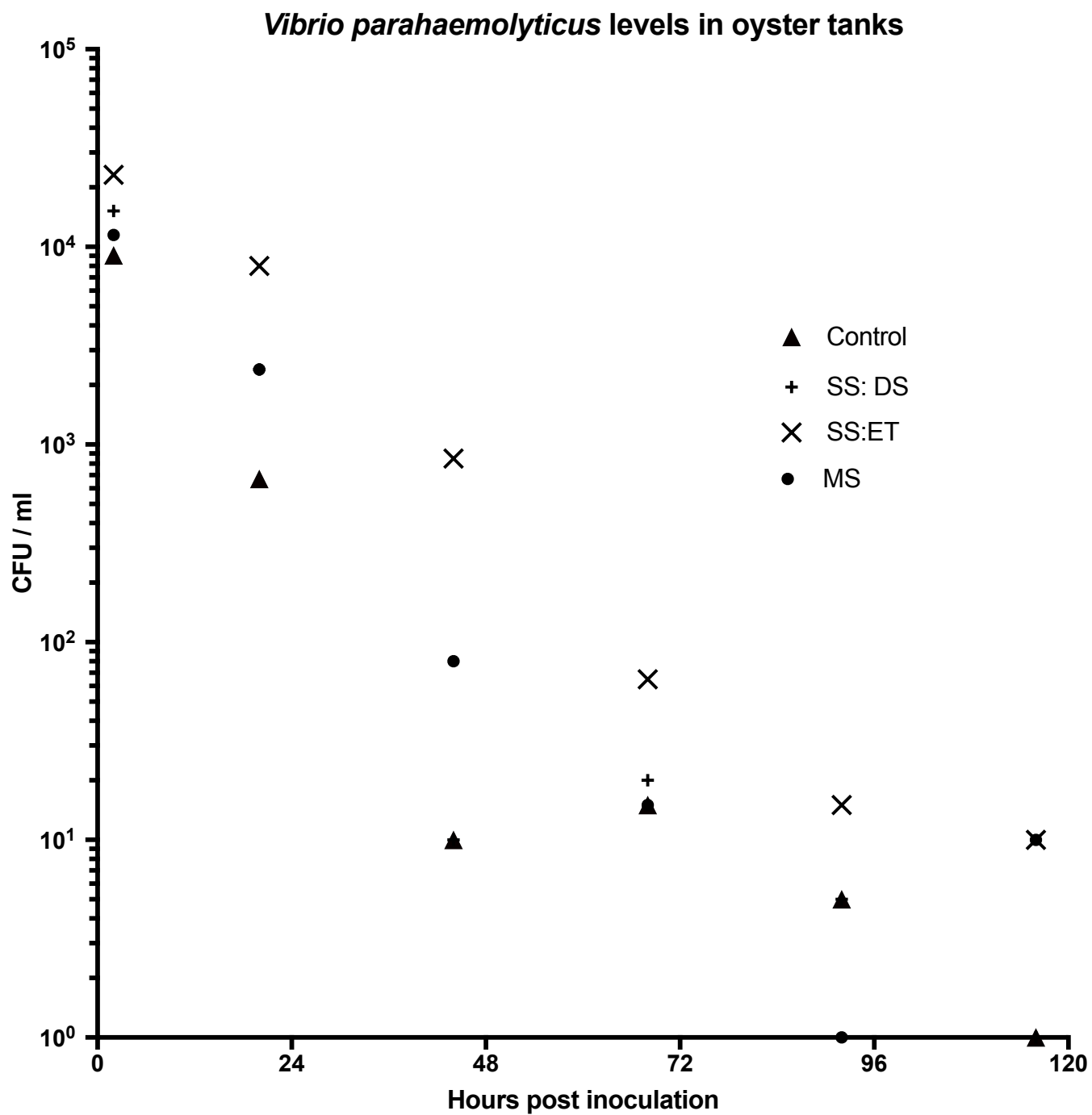

Supplement: SUPPLEMENTARY FIGURE S1 — Diagram of the experimental system setup. [file Data_Sheet_1.zip › FigS2.pdf]

Fig. S3

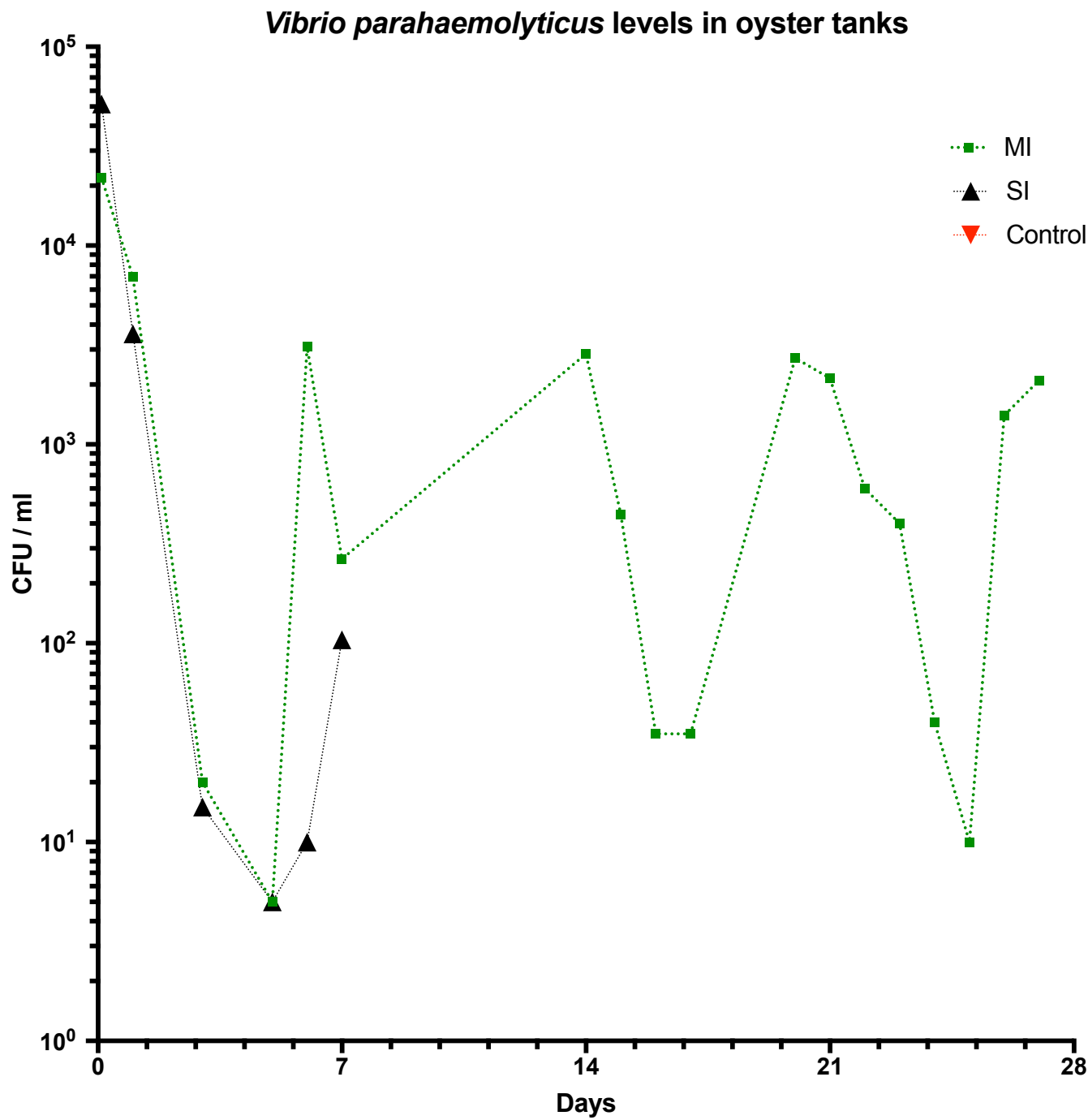

Supplement: SUPPLEMENTARY FIGURE S1 — Diagram of the experimental system setup. [file Data_Sheet_1.zip › FigS3.pdf]

Fig. S4

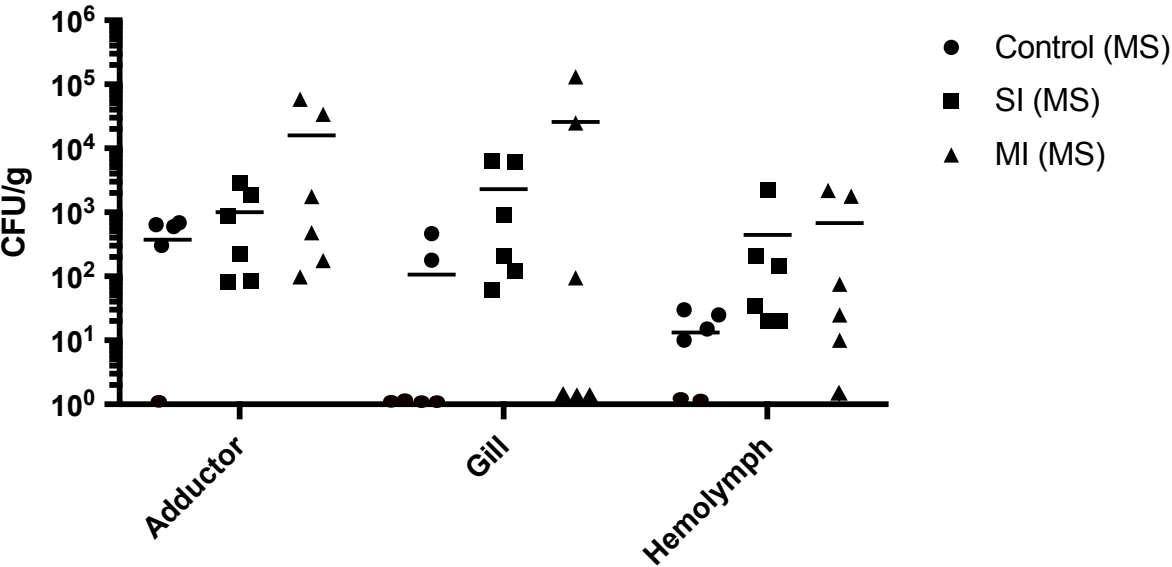

Supplement: SUPPLEMENTARY FIGURE S1 — Diagram of the experimental system setup. [file Data_Sheet_1.zip › FigS4.pdf]
